# Imaging Features and Clinical Characteristics of Granular Cell Tumors: A Single-Center Investigation

**DOI:** 10.3390/diagnostics15111336

**Published:** 2025-05-26

**Authors:** Hui Gu, Lan Yu, Yu Wu

**Affiliations:** 1 Department of Ultrasound, Union Hospital, Tongji Medical College, Huazhong University of Science and Technology, Wuhan 430022, China; celiagh@hust.edu.cn; 2 Department of Pathology, Union Hospital, Tongji Medical College, Huazhong University of Science and Technology, Wuhan 430022, China; 2011xh0806@hust.edu.cn

**Keywords:** granular cell tumor, imaging features, Fanburg–Smith criteria, star-like crack sign

## Abstract

**Background/Objectives:** Granular cell tumors (GCTs) are rare neurogenic tumors with Schwann cell differentiation. Although most are benign, 1–2% exhibit malignant behavior. The imaging features of GCTs remain poorly characterized due to their rarity and anatomic variability. This study aims to elucidate the manifestations of GCTs in multimodal imaging across different anatomic locations. **Methods:** We retrospectively analyzed 66 histopathologically confirmed GCT cases (2011–2024), assessing their clinical presentations, pathological characteristics, and imaging findings from ultrasound (*n* = 31), CT (*n* = 14), MRI (*n* = 8), and endoscopy (*n* = 15). Two radiologists independently reviewed the imaging features (location, size, morphology, signal/density, and enhancement). **Results:** The cohort (mean age: 42 ± 12 years; 72.7% female) showed tendency in location towards soft tissue (48.4%), the digestive tract (30.3%), the respiratory system (7.6%), the breasts (7.6%), and the sellar region (6.1%). Six cases (9.1%) were malignant. The key imaging findings by modality were as follows: Ultrasound: Well-circumscribed hypoechoic masses in soft tissue (96.1%) and irregular margins in the breasts (80%, BI-RADS 4B) were found. MRI: The sellar GCTs exhibited T1-isointensity, variable T2-signals (with 50% showing “star-like crack signs”), and heterogeneous enhancements. The soft tissue GCTs were T1-hypointense (75%) with variable T2-signals. CT: Pulmonary/laryngeal GCTs appeared as well-defined hypodense masses with mild/moderate enhancements. Endoscopy: Submucosal/muscularis hypoechoic nodules with smooth surfaces were found. Malignant GCTs were larger (mean: 93 mm vs. 30 mm) but lacked pathognomonic imaging features. Three malignant cases demonstrated metastases. **Conclusions:** GCTs exhibit distinct imaging patterns based on their anatomical location. While certain features (e.g., star-like crack signs) are suggestive, imaging cannot reliably differentiate benign from malignant variants. Histopathological confirmation remains essential to diagnosis, particularly given the potential for malignant transformations (at 9.1% in our series). Multimodal imaging guides the localization and biopsy planning, but clinical–radiological–pathological correlation is crucial for the optimal management.

## 1. Introduction

Granular cell tumors (GCTs) are neurogenic tumors characterized by Schwann cell differentiation. They comprise rounded or polygonal cells exhibiting reddish granules in the cytoplasm. Immunohistochemical analyses reveal the positive expression of certain nerve-specific markers, including Vimentin, S-100, and NSE [1]. Predominantly benign in nature, GCTs generally exhibit a favorable prognosis following surgical resection [2]. However, a minute fraction of patients with GCTs display malignant behavior, constituting approximately 1–2% of all GCT cases [3]. This subgroup often experiences suboptimal surgical outcomes, marked by a heightened risk of local recurrence and distant metastasis [4,5].

Clinically, granular cell tumors (GCTs) are uncommon entities, constituting a mere 0.5% of all soft tissue tumors [4]. Their occurrence is not restricted by age or anatomical site, as they manifest in various locations throughout the body. The existing literature indicates that GCTs tend to manifest in individuals aged 16 to 58 years, with approximately half of cases arising in the skin, subcutaneous, and submucosal tissues. Additionally, nearly one-third of cases localize in the tongue, while the remaining occurrences are distributed across the breasts, the digestive tract, the respiratory tract, the urogenital system, and other anatomical regions [6].

Given their rarity, the available literature on GCTs predominantly comprises small case series or individual case reports [7,8,9,10,11,12,13,14,15]. Notably, there is a paucity of imaging documentation, and details pertaining to the imaging features are often limited. In an effort to address this gap, our study comprehensively reviewed the complete dataset of 66 confirmed GCT cases obtained through pathology at a single center spanning from 2011 to 2024. Our analysis, incorporating multimodal imaging manifestations and complemented by a thorough examination of the existing literature, aims to enhance our comprehension of the imaging characteristics associated with this condition.

## 2. Material and Methods

### 2.1. Patients

Between 2011 and 2024, our institution diagnosed and pathologically confirmed 66 cases of granular cell tumors. Following the Fanburg–Smith criteria [2], the tumors were categorized into benign, atypical, and malignant classifications. Among these, 31 cases underwent ultrasonography, 14 cases underwent CT examination, 8 cases underwent MR examination, and 15 cases underwent gastroscopy; among the latter, 12 cases underwent ultrasound gastroscopy.

This study was approved by the Ethics Committee of Union Hospital at Huazhong University of Science and Technology, and its methods were applied in accordance with the approved guidelines. The retrospective nature of this study and the anonymization of the patient data led to a waiver of patient consent.

### 2.2. Examination

GE LOGIQ E9, Philips EPIQ 7 and Mindray DC80 color Doppler ultrasound instruments were used for the ultrasound examinations, and the probe frequency was 6~15 MHz. The CT examinations used a United States GE LightSpeed VCT 64-slice spiral CT scanner or a Siemens SOMATOM Drive 64-slice dual-source CT scanner. The MR examinations were performed using a GE Signa excite HD 1.5 T MR full-body scanner or a Philips Ingenia 3.0 T MR scanner.

### 2.3. Analysis of the Images

Images were analyzed by two radiologists independently with 11 and 5 years of work experience, respectively, using a picture archiving and communications system. Any disagreements were resolved through a consensus. The tumor locations; sizes; shapes; margins; density differences and enhancement features on CT; signal intensity on T1WI and T2WI; and echogenicity and internal blood flow signals on ultrasound were reviewed.

## 3. Results

### 3.1. Clinical Findings

Table 1 presents the clinical characteristics of the study cohort. With the exception of two pediatric cases, all individuals diagnosed were adults. The mean age of the patients was 42 ± 12 [SD] years (range: 7–69 years), and their median age was 45 years. The majority of the patients were female (72.7%), while male patients constituted 27.3% of the cohort. Table 2 shows the localization of the GCTs in this study. The tumor distribution across anatomical locations included soft tissue (32, 48.4%), the digestive system (20, 30.3%), the respiratory system (5, 7.6%), the breasts (5, 7.6%), and the sellar region (4, 6.1%). The most common clinical symptoms of soft tissue tumors are palpable painless masses and the presence of orbital masses, with decreased vision, pain, and eye movement disorders. In the digestive system, depending on the location of the tumor, such as in the tongue, esophagus, stomach, or colon, it often presents as choking, abdominal pain, bloating, and/or diarrhea, and in the tongue, it usually presents as a painless lump. In the respiratory system, vocal cord masses mostly manifest as hoarseness, and larynx and lung masses manifest as coughing and sputum production, chest tightness, or choking after eating. Breast lesions appear as lumps that are palpable or found on physical examination. Sellar region masses present with decreased vision, dizziness or headaches, emaciation, and fatigue. In this study, 2 cases were atypical (3.0%), 58 cases were benign (87.9%), and the remaining 6 cases were malignant (9.1%), 1 of which occurred in the back, 2 of which occurred in the thigh muscles, 2 of which occurred in the lungs, and 1 of which occurred in the orbit; 1 of the cases developed lung metastases a few years later.

### 3.2. Imaging Findings

#### 3.2.1. Soft Tissue

**Figure 1:**
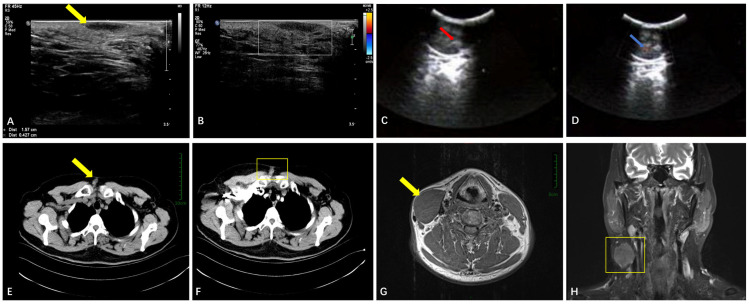
(**A**,**B**) A left perianal benign GCT, which was morphologically regular and smooth (see the yellow arrow) on ultrasound images and in which there was no colored blood flow signal; (**C**,**D**) a malignant GCT in the back with a regular morphology, clear borders, uneven internal echo (see the red arrow), and a colored blood flow signal on ultrasound images (blue arrow); (**E**,**F**) a CT scan and an enhanced image of a right anterior GCT in the chest wall showing a uniform density of nodular shadows (yellow arrow) and the enhanced scan showing enhancements (yellow border); (**G**,**H**) MRI images of a GCT in the right sternocleidomastoid muscle showed an equally weighted signal on T1WI (yellow arrow) and an uneven hyperintense shadow on T2WI (yellow border). (**E**,**F**) share a bar, (**G**,**H**) share a bar.

The average age of the 32 patients with soft tissue GCTs was 39 years old (range: 15~57 years), 22 of whom were female and 10 of whom were male. A total of 26 cases were benign, and the maximum diameter of the tumors was 11~90 mm, while the average diameter was 30 mm. Of these cases, 23 cases were performed using ultrasound, and 22 cases showed a regular morphology, low echo with clear boundaries and uniform internal echoes, no internal calcification, and no attenuation in posterior echoes (Figure 1A,B), while only 1 case had an irregular morphology and unclear borders. Low blood flow was seen in the mass in three cases, and no blood flow was seen in the rest. A CT examination was performed in 8 cases, and nodular shadows with clear boundaries (Figure 1E) and moderate enhancement were seen (Figure 1F). MR examinations were performed in four cases (Figure 1G,H); three of these lesions (3/4) showed as hypointense in T1-weighted sequences, while one case (1/4) was isointense in T1-weighted sequences. Two of these lesions (2/4) showed as hyperintense in T2-weighted sequences, and two cases showed mixed signal shadows. Four cases were malignant, one in the back, two in the lower limbs, and one in the orbit, and the maximum diameter of the tumors was 35~190 mm, while the average diameter was 93 mm. CT examination of the orbit showed shadows in the intraorbital soft tissue, and ultrasonography in the remaining three cases showed clear boundaries and regular morphologies (Figure 1C,D), with visible blood flow in one case and no blood flow in the other two cases, which appeared similar to ultrasound images of benign soft tissue GCTs. These findings are synthesized in Table 3. Two cases were atypical, one in the chest wall and one in the lower limbs. After 2~60 months of follow-up of these 32 cases, 1 case located in the back recurred after three years, while the rest did not recur.

#### 3.2.2. The Digestive System

**Figure 2:**
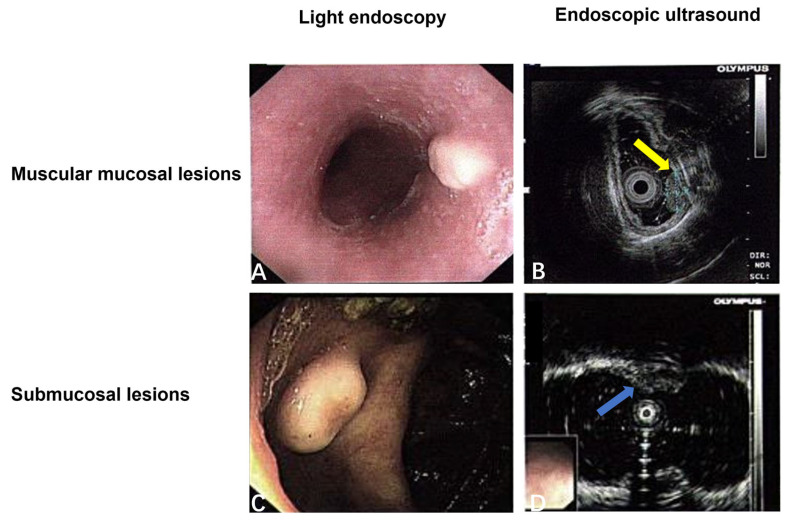
(**A**,**B**) Mucosal muscularis lesions under white light endoscopy and endoscopic ultrasound (yellow arrow); (**C**,**D**) submucosal lesions under white light endoscopy and endoscopic ultrasound (blue arrow).

The average age of the 20 patients with digestive system GCTs was 42 years old (range: 7~69 years); 15 cases were female and 5 cases were male. All 20 cases were benign (5 cases were located in the tongue, 10 cases were distributed in the esophagus, 2 cases occurred in the stomach, and 3 cases occurred in the colon); the maximum diameter of the tumors was about 3~8 mm, and their average diameter was 1 mm. Digestive endoscopy was performed in all cases except for those that occurred in the tongue. In white light endoscopy, they generally presented as raised lesions with a smooth surface (Figure 2A,C). Twelve cases of endoscopic ultrasounds were performed, and the results showed that five cases (5/12, 41.7%) were located in the muscular layer and seven cases (7/12, 58.3%) were located under the mucosa, with a clear boundary from the surrounding tissues (Figure 2B,D). These findings are synthesized in Table 3. Twenty cases were followed up (2~60 months), and there was no recurrence.

#### 3.2.3. The Respiratory System

**Figure 3:**
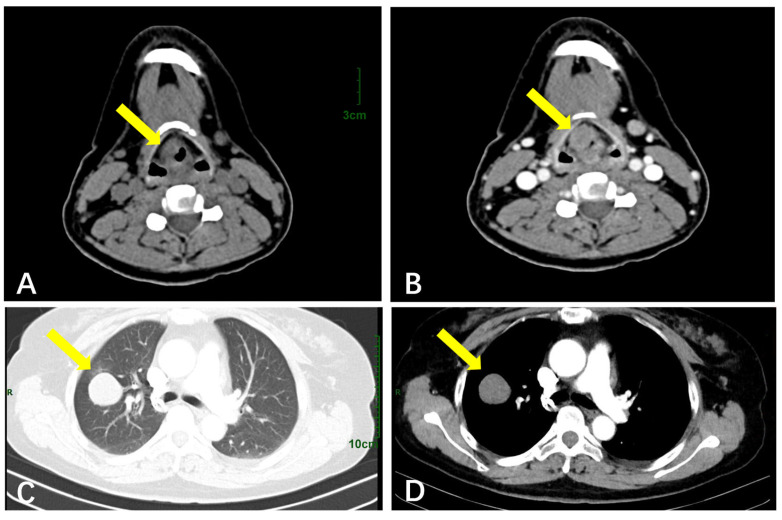
(**A**) Noncontrast CT scan showed a benign mass in the right larynx with well-defined soft tissue density opacity (yellow arrow); (**B**) CT with contrast showed enhancement within the mass; (**C**) noncontrast CT scan showed a malignant mass in the lung with clear borders (yellow arrow), and there was no significant difference in the image compared with the benign mass; (**D**) CT with contrast showed enhancement within the mass. (**A**,**B**) share a bar, (**C**,**D**) share a bar.

The average age of five patients with respiratory GCTs was 48 years old (range 38~52 years); four were female, and one was male. Three cases were benign, the maximum diameter of their tumors was 3~23 mm, and the average was 12 mm. CT showed a round or oval soft tissue mass shadow with clear boundaries and a lobulated or irregular morphology, and mild–moderate enhancement could be seen on enhanced CT scanning (Figure 3A,B). Two cases were malignant, with a maximum diameter of 35~65 mm and an average diameter of 50 mm, and the CT findings had no obvious specificity compared with benign GCTs (Figure 3C,D). These findings are synthesized in Table 3. Five cases were followed up (2~60 months), and there was no recurrence.

#### 3.2.4. Breast GCTs

**Figure 4:**
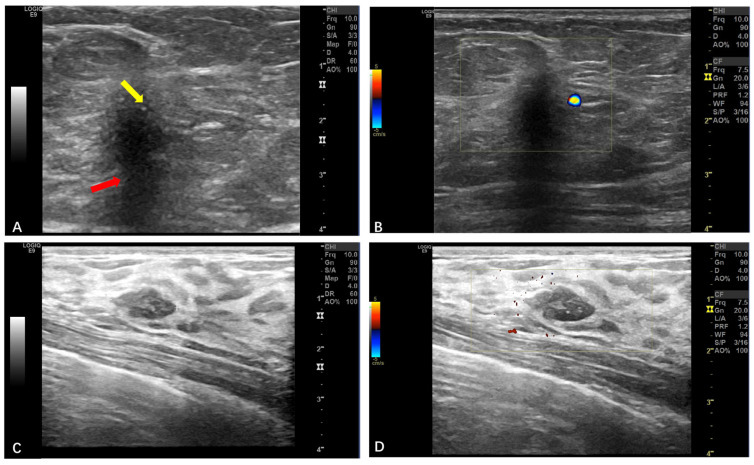
(**A**,**B**) The margins of the lateral glands of the right breast showed a hypoechoic area, an irregular morphology, untidy margins, posterior echo attenuation (red arrow), and strong echogenic spots inside (yellow arrow); there were no obvious blood flow signals. A lateral parenchymal mass of the right mammary gland of BI-RADS class 4B. (**C**,**D**) Hypoechoic nodules in the upper inner left breast with clear boundaries and uniform internal echoes without an obvious blood flow signal. Parenchymal nodules in the left breast, BI-RADS class 3.

The average age of the five patients with breast GCTs was 47 years old (range: 36~59 years); all of them were female cases; the pathological results were all benign; the maximum diameter of their tumors was 5~29 mm, and the average diameter was 14 mm; and the ultrasound manifestations in these four cases (4/5) were all hypoechoic masses with an irregular morphology and smooth edges, a non-parallel orientation, and posterior echo attenuation. Strong echo spots were seen in two cases (Figure 4A,B), and the conclusions suggested BI-RADS class 4B, while only one case (1/5) showed a hypoechoic mass with smooth edges and clear boundaries (Figure 4C,D), and the conclusions were suggestive of BI-RADS class 3. Color Doppler ultrasound showed no obvious blood flow signals in the internal structures of these five masses. These findings are synthesized in Table 3. After follow-up of the five cases (2~60 months), there was no recurrence.

#### 3.2.5. The Sellar Region

**Figure 5:**
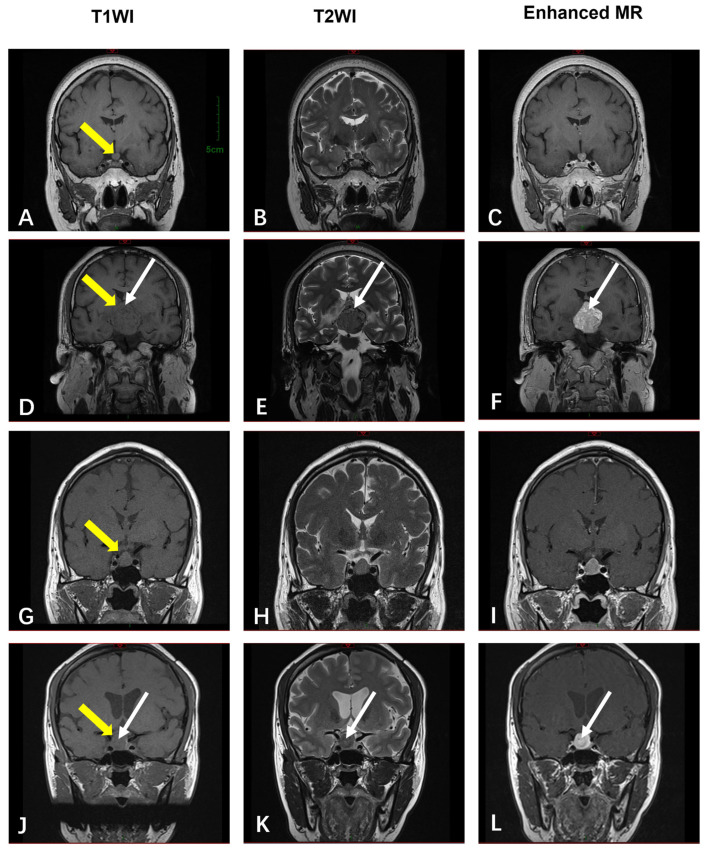
MRI images of 4 patients with sellar GCTs. (**A**,**D**,**G**,**J**) show that the 4 cases were isointense in T1WI (yellow arrows indicating the location of the lesions); (**B**,**E**,**H**,**K**) show variable signals in T2WI, ranging from isointense signals to low signals or slight hyperintensity; (**C**,**F**,**I**,**L**) show enhancement in all 4 cases. Note that within the masses, there are star-like cracks (white arrows in **D**–**F**,**J**–**L**) that show hypointensity on T1WI and hyperintensity on T2WI. The star-like crack is enhanced after contrast. Other subgraphs in Figure 5 and subgraph A share one scale bar.

The average age of the four patients with sellar GCTs was 49 years old (range: 33~57 years), of whom two were female and two were male. All four cases were benign, with a maximum diameter of 8~46 mm and an average diameter of 22 mm, and all four cases were examined using MRI, in which these tumors showed equal signals in the T1-weighted sequences (Figure 5A,D,G,J) and variable signals in the T2-weighted sequences, ranging from isointensity to low signals or slight hyperintensity (Figure 5B,E,H,K). The contrast scans showed uniform enhancement in two cases and non-uniform enhancement in two cases (Figure 5C,F,I,L). Two cases showed star-like cracks, with low signals in T1WI and high signals in T2WI, which showed significant enhancement after comparison. These findings are synthesized in Table 3. After follow-up of the four cases (2~60 months), there was no recurrence.

## 4. Discussion

In this study, we reviewed 66 cases of GCTs diagnosed at our center over the past 13 years. The age of onset ranged from 7 to 69 years, with an average age of 42 ± 12 [SD] years and a median age of 45 years. GCTs are typically female-predominant, ranging from 1.8 to 2.9:1 [16,17,18], and this predilection was confirmed in our study (2.7:1). The most common site of GCTs was the soft tissue (48.4%), followed by the digestive system (30.3%), the respiratory system (7.6%), the breasts (7.6%), and the sellar region (6.1%). The tumors themselves did not exhibit specific clinical symptoms, but compression-related manifestations appeared depending on the tumor’s location.

Generally, a GCT appears as homogeneous gray-white or brown nodules without a capsule. Most GCTs are poorly defined and infiltrate the surrounding tissues, including dermal collagen fibers, adipose tissue, and skeletal muscle [13]. Regardless of location, the microscopic characteristics of benign GCTs are very consistent. They are composed of large polyhedral cells arranged into sheets, nests, and strips, with small nuclei, uniform staining, and a rich granular eosinophilic cytoplasm [19]. The stroma contains abundant collagen fibers, which can become fibrotic in cases with a long disease course. GCTs are believed to derive from the nerve tissue due to their positive staining for S-100 and neuron-specific enolase [20]. Traditional GCTs are benign tumors, with less than 2% of cases developing into malignant tumors [3,21]. Malignancy is diagnosed through a combination of histological findings (including cell pleomorphism and increased mitotic activity) and clinical malignant behavior, according to the Fanburg–Smith criteria [1]. GCTs were subdivided into malignant, atypical, or benign based on the presence of the following six histological features: necrosis, spindling of the tumor cells, vesicular nuclei with large nucleoli, an increased mitotic rate (>2 mitoses/10 high-power fields at 200× magnification), an increased nuclear-to-cytoplasmic ratio, and nuclear pleomorphism. Tumors were classified as malignant if there were three or more of these features, atypical if there were only one to two of these features, and benign if there were none of these features or focal nuclear pleomorphism was present. Clinically, if a lesion is large, grows rapidly, or shows evidence of distant spread, it should be highly suspected as malignant.

For small nodules in the skin or the oral cavity, imaging examinations are typically not conducted prior to biopsy. However, imaging is generally necessary for tumors located in the gastrointestinal tract, breasts, soft tissues, or other atypical sites.

Ultrasound examinations are primarily used for GCTs in the breasts and soft tissues. In the breast cases, most of the tumors (4/5, 80.0%) in our series exhibited distinctive ultrasonic features: irregular edges, a non-parallel orientation, low or heterogeneous internal echoes, and posterior acoustic attenuation. These findings are consistent with those reported by Meani F et al. [5,22,23,24]. The irregular edges reflect the infiltrative growth pattern of the GCTs into the surrounding adipose tissue, while the internal echo indicates the dense cellular composition and fibrous matrix of the tumor. Notably, all five mammary GCT cases in our study demonstrated an absent intralesional blood flow on color Doppler imaging. While previous reports have described non-specific vascular patterns in mammary GCTs, ranging from hypovascularity to detectable vascularity, our findings suggest a higher prevalence of avascularity in this cohort. The soft tissue GCTs and breast GCTs demonstrated mostly different ultrasound characteristics. Of the soft tissue cases, 26 cases (23 benign and 3 malignant) were examined with ultrasound; 25 of these lesions (25/26, 96.1%) had clear boundaries and parallel growth; and the blood flow signals in CDFI of the GCTs were typically sparse. Currently, there has been no research into the use of ultrasound imaging systems for soft tissue granular cell tumors. However, the limited sample size (*n* = 23) at our single institution may constrain the generalizability of these findings. Among them, there were three cases of malignant GCTs, and in our study, the malignant soft tissue GCTs did not show the morphology of conventional malignancies, such as unclear boundaries with the surrounding tissues and an irregular morphology. Generally, GCTs in the breasts and soft tissues can present with imaging features of either clear boundaries or invasive growth, mimicking the appearance of benign lesions or invasive cancer [16,25]. In fact, both in the upper mammary gland and in the extramammary soft tissue, GCTs grow along the fascia; because the female breast is located in the superficial fascia in front of the pectoralis major muscle and there is a suspensory ligament supporting the breast inside, the characteristics of GCTs growing along the fascia are particularly complex in the mammary region, and hyperplasia of the connective tissue caused by the tumor causes the surrounding tissue to shrink, the skin is involved and is dimpled, and deep muscle invasion is involved and tumors can adhere to the pectoral muscle, which means that female breast GCTs tend to have malignant sonographic features, characterized by an irregular morphology and unclear borders [26]. However, in soft tissues such as the limbs or the trunk, GCTs are restricted by the muscles themselves from growing in the myofascial direction, so they appear in parallel positions. Imaging-guided biopsy is instrumental in differentiating between these lesions.

MRI is commonly used to diagnose GCTs in the brain, throat, and soft tissues. GCTs of the brain and larynx typically present as solid masses with clear boundaries. In our study, all four cases of intracranial GCTs were located in the sellar region. These tumors were isointense in T1-weighted sequences and showed as variable signals in T2-weighted sequences, ranging from isointense to hypointense or slightly hyperintense. Enhanced scans revealed uniform enhancement in two cases and inhomogeneous enhancement in two cases. Notably, two cases demonstrated patchy, slightly hyperintense signals in the T2WI sequences, which showed significant enhancements post-contrast. This pattern aligns with the “star-like crack sign” in T2WI reported by Han et al. [27], suggesting it may represent a typical imaging feature. In contrast, the MRI findings for the soft tissue GCTs are not entirely consistent with those for intracranial GCTs. In T1-weighted imaging, three of these lesions (3/4) showed as hypointense, while only one case showed as isointense. In the T2-weighted sequences, two of these lesions (2/4) showed as hyperintense, and two cases showed mixed signal shadows.

CT scanning is commonly utilized to diagnose GCTs in the respiratory system, throat, and soft tissues. In our series, three cases of lung GCTs were identified: one was a larynx GCT, one appeared in the vocal cords, and one was a metastatic GCT originating from a malignant granular cell tumor in the right shoulder. CT imaging revealed a soft tissue density mass with well-defined margins. The GCTs typically appeared as slightly hypodense compared to the adjacent muscle tissue. They were characterized by a lobulated or irregular shape with a homogeneous internal density and no evidence of cystic necrosis or calcification. The enhancement on CT was either mild or heterogeneous, consistent with the findings reported in the literature [28]. Additionally, although rare, there have been reports of GCTs in the lungs occurring concurrently with lung adenocarcinoma [29].

Gastrointestinal GCTs are typically identified during digestive endoscopy. In white light endoscopy, these tumors often present as convex lesions with smooth surfaces, and large tumors may exhibit surface ulcers. Endoscopic ultrasonography reveals that GCTs originate from the muscularis mucosa or the submucosa, displaying uniform hypoechoic or slightly hyperechoic changes with well-defined margins. These results are in accordance with the findings reported by Finer et al. [30], who observed similar characteristics in gastrointestinal GCTs.

In our series, we identified six cases of malignant GCTs. These tumors were in various sites: two in the muscularis of the lower limbs, one in the back, two in the lungs, and one in the orbit. Lymph node or lung/liver metastases were observed in three of these cases. Malignant GCTs are characterized by large tumor volumes and rapid growth. Of the six cases, one lower limb muscle GCT exhibited invasive imaging features, while the remaining five presented as well-defined solid masses.

As summarized in Table 3, GCTs present with varied imaging characteristics depending on their location. Most cases are characterized by benign tumors, but these tumors can also exhibit aggressive growth patterns, especially when located in the adipose tissue, skeletal muscle, or dermal collagen fibers, often mimicking malignant lesions. However, even if there are signs of malignancy, the pathological results may not necessarily be malignant. While imaging techniques, including ultrasound, CT, and MRI, can suggest the presence and nature of GCTs, they lack the specific features for a definitive diagnosis. Therefore, a comprehensive diagnostic approach combining clinical evaluation and pathological examination is essential for accurate identification, differentiation from other malignancies, and effective management.

## 5. Conclusions

GCTs exhibit site-dependent imaging variability; however, definitive differentiation between benign and malignant variants relies on histopathological confirmation. While most benign GCTs demonstrate well-circumscribed margins, the overlapping imaging characteristics with their malignant counterparts necessitate caution. Complete surgical resection remains the cornerstone of benign GCT management, whereas malignant cases require long-term surveillance due to their metastatic potential. Multimodal imaging serves critical roles in lesion localization and biopsy guidance, but a final diagnosis mandates an integrated clinical–radiological–pathological correlation.

## Figures and Tables

**Table 1:** Clinical characteristics of the 66 patients.

**Age**	**Years**
range	7–69
median, mean ± SD	45, 42 ± 12
**Gender**	
female	48 (72.7%)
male	18 (27.3%)
**Lesion Location**	**Presenting Symptoms**
soft tissue	palpable painless masses
orbit	decreased vision, pain, and eye movement disorders
digestive system	choking, abdominal pain, bloating, diarrhea
respiratory system	hoarseness, coughing, sputum production, chest tightness, or choking after eating
breast	palpable lumps
sellar region	decreased vision, dizziness or headache, emaciation, and fatigue
**Pathological Nature**	**Number of Cases, (%)**
benign	58 (87.9%)
atypical	2 (3.0%)
malignant	6 (9.1%)

**Table 2:** Localization of GCTs in this study.

Lesion Location	Number of Cases, (%)
**soft tissue**	**32 (48.4%)**
upper limbs	2 (3.0%)
lower limbs	4 (6.1%)
neck	2 (3.0%)
chest wall	4 (6.1%)
abdominal wall	8 (12.1%)
back	5 (7.6%)
crissum	1 (1.5%)
axilla	1 (1.5%)
orbit	3 (4.5%)
umbilicus	1 (1.5%)
groin	1 (1.5%)
**digestive system**	**(20, 30.3%)**
tongue	5 (7.6%)
esophagus	10 (15.2%)
stomach	2 (3.0%)
colon	3 (4.5%)
**respiratory system**	**(5, 7.6%)**
larynx	1 (1.5%)
vocal cords	1 (1.5%)
lung	3 (4.5%)
**breasts**	**(5, 7.6%)**
**sellar region**	**(4, 6.1%)**

**Table 3:** Summary of key imaging features of GCTs by anatomical location.

Location	Imaging Modality	Key Features	Malignant Indicators
**Soft Tissue**	Ultrasound (*n* = 26)	Well-circumscribed hypoechoic masses (*n* = 25, 96.1%); sparse blood flow.	Larger size (mean: 93 mm); rare irregular margins.
	CT (*n* = 9)	Well-defined hypodense nodules (*n* = 9, 100%); moderate enhancement	N/A
	MRI (*n* = 4)	T1-hypointense (*n* = 3, 75%) or T1-isointense (*n* = 1, 25%); variable T2-signals (hyper-/mixed).	N/A (all benign)
**Digestive System**	Endoscopy/EUS (*n* = 15)	Submucosal/muscularis hypoechoic nodules; smooth surface (*n* = 15, 100%).	N/A (all benign)
**Respiratory System**	CT (*n* = 5)	Round/oval hypodense masses; lobulated contours; mild–moderate enhancement	A larger size (mean: 50 mm); no pathognomonic signs.
**Breasts**	Ultrasound (*n* = 5)	Irregular margins; a non-parallel orientation; posterior shadowing (BI-RADS 4B) (*n* = 4, 80%)	N/A (all benign)
**Sellar Region**	MRI (*n* = 4)	T1-isointense; T2-variable (“star-like crack signs” in 50%); heterogeneous enhancement in 50%	N/A (all benign)

EUS: endoscopic ultrasound; N/A: not applicable (no distinct features in this cohort).

## Data Availability

The data presented in this study are available on request from the corresponding author. The data are not publicly available due to privacy.
